# Efficacy of stem cell therapy for pulmonary arterial hypertension: a systematic review and meta-analysis of preclinical studies

**DOI:** 10.1186/s13287-019-1162-8

**Published:** 2019-02-13

**Authors:** Xian-Fei Ding, Huo-Yan Liang, Bo Yuan, Li-Feng Li, Tian Wang, Quan-Cheng Kan, Le-Xin Wang, Tong-Wen Sun

**Affiliations:** 1 General ICU, Henan Key Laboratory of Critical Care Medicine, The First Affiliated Hospital of Zhengzhou University, Zhengzhou, 450052 China; 2 Biotherapy Center, The First Affiliated Hospital of Zhengzhou University, Zhengzhou, 450052 China; 3 Department of Pharmacy, The First Affiliated Hospital of Zhengzhou University, Zhengzhou, 450052 China; 4 School of Biomedical Sciences, Charles Sturt University, Wagga Wagga, NSW 2650 Australia

**Keywords:** Pulmonary hypertension, Stem cells, Animal model, Therapy, Meta-analysis

## Abstract

**Background:**

Despite significant progress in drug treatment, the prognosis of patients with advanced pulmonary arterial hypertension (PAH) remains extremely poor. Many preclinical studies have reported the efficacy of stem cell (SC) therapy for PAH; however, this approach remains controversial. The aim of this systematic review and meta-analysis is to assess the potential efficacy of SC therapy for PAH.

**Methods:**

The Medline, EMBASE, Cochrane Library, and Web of Science databases were searched from inception to August 12, 2018. Preclinical studies that evaluated the use of SC therapy for PAH were included. The primary outcome was pulmonary haemodynamics, as assessed by measurement of the right ventricular systolic pressure (RVSP), mean pulmonary arterial pressure (mPAP), and/or mean right ventricle pressure (mRVP). The secondary outcomes included the weight ratio of the right ventricle to the left ventricle plus septum (RV/LV+S), the right ventricle to body weight ratio (RV/BW), the percentage of pulmonary arteriole area index (WA), and/or the percentage of medial wall thickness of the pulmonary arteriole (WT). The quality of outcomes was evaluated using the SYstematic Review Centre for Laboratory animal Experimentation (SYRCLE) bias risk tool. The inverse-variance method with random-effects modelling was used to calculate pooled weighted mean differences (WMDs) and 95% CIs. Statistical analysis was performed with STATA 14.0.

**Results:**

Twenty-eight eligible articles (722 animals) were included. SC therapy reduced the pooled WMDs (95% CIs) of RVSP, mPAP, mRVP, RV/LV+S, RV/BW, WA, and WT for animals with PAH, with values of − 14.12 (− 14.63, − 13.61), − 11.86 (− 12.35, − 11.36), − 17.33 (− 18.10, − 16.56), − 0.10 (− 0.10, − 0.09), 0.23 (0.21, 0.24), − 13.66 (− 15.71, − 11.62), and − 7.96 (− 7.99, − 7.93), respectively.

**Conclusions:**

SC therapy is effective for PAH in preclinical studies. These results may help to standardise preclinical animal studies and provide a theoretical basis for clinical trial design in the future.

**Systematic review registration:**

PROSPERO (http://www.crd.york.ac.uk/PROSPERO).

**Electronic supplementary material:**

The online version of this article (10.1186/s13287-019-1162-8) contains supplementary material, which is available to authorized users.

## Background

Pulmonary arterial hypertension (PAH) is a progressive chronic disease with a high mortality rate [1], and the median survival of patients with idiopathic PAH was estimated to be 2.8 years [2]. This disease is characterised by progressively increasing PAH and vascular remodelling [3], which ultimately leads to right heart failure and death [4]. The pulmonary vasculature is also remodelled, with increased pulmonary vascular resistance and over-proliferation of pulmonary artery endothelial cells [5, 6]. In recent decades, the treatment of PAH has progressed significantly, as a deeper understanding of the underlying pathogenesis has been achieved [7–12]. However, the mortality of PAH remains high [12, 13]. Therefore, there is a considerable unmet medical need in the management of PAH.

Recently, cell-based gene therapies have attracted increasing interest due to their beneficial roles in ameliorating the progression of PAH [14–16]. Stem cells (SCs) are multipotent progenitor cells, and mesenchymal SCs are the preferred seed cells for cell-based therapy because of their strong expansion ability in culture, their reproducible potential, and their capacity to differentiate into bone, cartilage, muscle, or vascular smooth muscle cells, as well as other connective tissues [17–19]. The ultimate goal of SC therapy is to inhibit pulmonary vascular remodelling and excessive cell proliferation, slowing the development of pulmonary hypertension and the occurrence of right heart failure to achieve clinical improvement of cardiopulmonary function without severe adverse effects.

Many animal studies of PAH have been reported, with heterogeneous designs and conflicting outcomes. Nevertheless, preclinical studies are needed to assess the risk of new therapies and to predict safety, feasibility, and efficacy. Moreover, such studies can offer guidance concerning unresolved issues related to clinical cell therapy (i.e., the choice of cell type and dose, the route and timing of delivery, and follow-up after cell transplantation). Thus, we performed a systematic review of the pertinent literature, including a quantitative meta-analytical pooling of the data, to assess the effects of SC therapy on animals with PAH.

## Methods

The author declares that all supporting data are available within this article and its online supplementary files. The study protocol is registered through PROSPERO (http://www.crd.york.ac.uk/PROSPERO), and the registration number is CRD42018103854, which can be found online at https://www.crd.york.ac.uk/PROSPERO/display_record.php?RecordID=103854.

### Data sources and search strategies

This meta-analysis followed the Preferred Reporting Items for Systematic Reviews and Meta-Analyses (PRISMA) criteria [20]. The PRISMA 2009 checklist is shown in Additional file 11: Table S1. A systematic literature search was conducted for animal studies on SC therapy and PAH that were published until 12 August 2018, using the Medline (www.ncbi.nlm.nih.gov/pubmed), EMBASE (www.embase.com), Cochrane Library (www.cochranelibrary.com), and Web of Science (http://apps.webofknowledge.com/WOS_GeneralSearch_input.do?) databases. The detailed search strategy is shown in Additional file 12: Table S2. The search was limited to English. We also hand-searched the references of the included articles. Literature searches were performed independently by Huo-Yan Liang and Bo Yuan.

### Eligibility criteria

Studies were considered suitable for inclusion in this meta-analysis if (1) all PAH animal models were subjected to SC treatment (all species and sexes), (2) saline-treated PAH animal models were used as controls, and (3) the studies included data on one or more outcomes, such as right ventricular systolic pressure (RVSP), mean pulmonary arterial pressure (mPAP), mean right ventricle pressure (mRVP), the weight ratio of the right ventricle to the left ventricle plus septum (RV/LV+S), the right ventricle to body weight ratio (RV/BW), the pulmonary arteriole area index (WA), and the wall thickness of the pulmonary arteriole (WT). The exclusion criteria were as follows: (1) all inclusion criteria were not fulfilled, (2) the animals had comorbidities, (3) the animal models did not have PAH, (4) animal models with PAH did not receive SC treatment, (5) the study was a case study, a crossover study, or a study without a separate control group, (6) the study was a duplicate report, or (7) weighted mean differences (WMDs) with 95% CIs were not provided or could not be calculated.

### Study selection and data extraction

The titles and/or abstracts of studies retrieved using the search strategy and studies from additional sources were screened independently by Huo-Yan Liang and Bo Yuan to identify studies that potentially met the inclusion criteria outlined above. Key data interpretation was conducted independently by Li-Feng Li and Tian Wang. Any differences were resolved through discussions with Quan-Cheng Kan and Le-Xin Wang. The extracted information was as follows: author (year of publication), animal characteristics (species, gender, sample size, and model), intervention characteristics (source, dosage, route, and timing of SC therapy), follow-up (observation time of outcomes after SC administration), and measures relevant to our primary and secondary outcomes.

### Assessment of risk of bias

The risk of bias of eligible studies was assessed using the SYstematic Review Centre for Laboratory animal Experimentation (SYRCLE) bias risk tool [21]. The SYRCLE tool was adapted from the Cochrane Risk of Bias Tool to assess methodological quality using criteria specific to animal studies. The items in this tool include assessments for selection bias (sequence generation, baseline characteristics, and allocation concealment), performance bias (random housing and blinding), detection bias (random outcome assessment and blinded outcome assessment), attrition bias (completeness of outcome data), and reporting bias. For each included study, results of no, yes, and unclear risk of bias were scored as high, low, and unclear risk of bias, respectively.

### Primary and secondary outcomes

The current gold standard for the diagnosis and evaluation of clinical PAH is direct pulmonary haemodynamic measures, as assessed by measurement of RVSP, mPAP, and mRVP through right heart catheterization. RV remodelling is a precursor of right heart failure and is characterised by decreased function and dilatation of the RV, and this change is strongly correlated with prognosis and survival in PAH patients [22]. We collected morphometric data on RV remodelling expressed as the RV/LV+S and RV/BW. In addition, we also collected data from other non-invasive measures obtained by echocardiography to evaluate cardiac structure and pulmonary haemodynamics, such as WA and WT. In this meta-analysis, the primary outcomes included RVSP, mPAP, and mRVP after SC administration. Secondary outcomes included RV/LV+S, RV/BW, WA, and WT.

### Statistical analysis

The estimated effect sizes of primary and secondary outcomes were determined by WMDs and 95% CIs. WMDs, an ideal measure for continuous data, were calculated by the mean (*M*), standard deviation (SD), and sample number (*N*) in each study. The pooled WMD of each study was conducted using a fixed-effects model (inverse-variance) or a random-effects model (I-V heterogeneity) to generate forest plots. In addition, the *Q* test and *I*^2^ test were used to measure heterogeneity, which was not considered significant if *P* > 0.1 or *I*^2^ < 50%. Potential sources of heterogeneity, if significant, were further investigated by stratified analysis and meta-regression. Begg’s funnel plot [23] and Egger’s linear regression [24] were used to assess potential publication bias. Funnel plots were visually assessed for asymmetry. For Egger’s tests, *P* < 0.1 was considered significant to confirm the presence of a small study size. All analyses were performed using Stata 14.0 statistical software (Stata Corp LP, College Station, Texas 77,845, USA, serial number: 401406267051). Differences for which *P* < 0.05 (two-sided) were considered statistically significant.

## Results

### Study selection

**Fig. 1:**
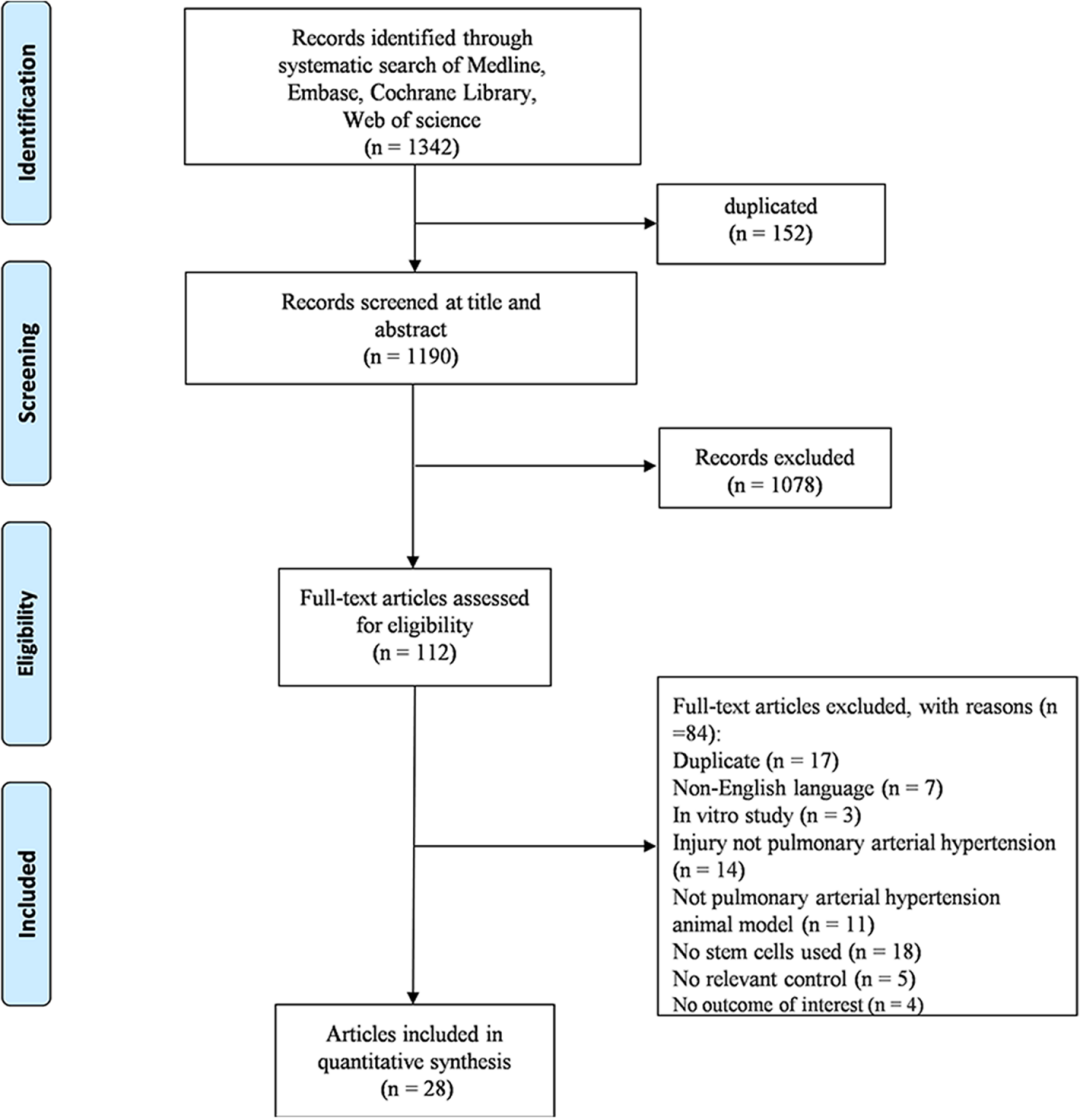
Flow diagram of study selection

Using our search strategy, we initially identified 1342 records, and 1190 articles remained after duplicates were removed. After preliminary screening by title and abstract, 112 articles reporting the therapeutic potential of SCs for PAH were isolated for full-text review. However, 28 articles [14, 15, 25–50] enrolling 722 animals were ultimately included in this meta-analysis after study selection (Fig. 1).

### Study characteristics

The number of included articles reporting RVSP, mPAP, mRVP, RV/LV+S, RV/BW, WA, and WT was16 [14, 26, 28–33, 39, 40, 42, 45, 48–50], 12 [25, 30, 38, 41, 44, 47, 50], 14 [27, 34–37, 42, 50], 29 [25–27, 29–31, 33–36, 39–41, 43–45, 47, 48, 50], 16 [14, 29, 34–37, 42, 48, 50], 5 [27, 43, 44], and 23 [26, 27, 32–37, 40, 42–44, 49, 50], respectively. The animal model used in most studies was induced by surgical operation or by 40–60 mg/kg monocrotaline (MCT) administered intraperitoneally, subcutaneously, or via tail vein injection. The majority of the animals were male, 6-week-old, Sprague Dawley rats, and weighed 180–220 g. Regarding the intervention characteristics of the SCs, they mostly originated from human or rat bone marrow, adipose-derived mesenchymal tissue, or human umbilical cord blood-derived mesenchymal tissue. The doses of the interventions ranged from 10^5^ to 10^7^ SCs, which were injected intravenously, intratracheally, or intraperitoneally at least 3 days after induction of the PAH model. The observation time of the primary and secondary outcomes after SC administration was at least 1 week. In addition, several articles contained more than one study [34–38, 44]. The main characteristics of the 28 articles involved in this meta-analysis are shown in Tables 1 and 2.

**Table 1:** General characteristics of preclinical studies investigating the efficacy of stem cells in models of PAH

Author (year)	Species, strain, sex	No. of controls	No. of treated animals	PAH model	SC source	SC route	SC dose	Timing of SC therapy after PAH	Follow-up (weeks)
Lim et al.(2016) [39]	Rat, SPF, M	5	5	MCT (60 mg/kg)	hBM	Tail vein	2.5 × 10^5^	14 days	2 weeks
Lee et al. (2017) [36]	Rat, SPD, M	4	6	MCT (60 mg/kg)	hUCB	Jugular vein	3 × 10^5^	7 days	1 week
									3 weeks
		4	6						
		4	4				3 × 10^5^	7 days	1 week
									3 weeks
		4	4						
Guo et al. (2013) [30]	Rat, SPD, M	10	10	MCT (60 mg/kg)	rBM	Jugular vein	5 × 10^6^	21 days	3 weeks
Luan et al.(2012) [42]	Rat, SPD, NR	20	20	Surgical operation	rBM	Sublingual vein	1–5 × 10^6^	28 days	2 weeks
Baber et al. (2006) [25]	Rat, SPD, M	10	10	MCT (60 mg/kg)	rBM	Intratracheal	3 × 10^6^	14 days	3 weeks
Chen et al. (2014) [27]	Rat, SPD, NR	7	7	MCT (50 mg/kg)	rBM	Intravenous	5 × 10^6^	21 days	2 weeks
Chen et al. (2016) [26]	Rat, Wistar, M	10	10	MCT (60 mg/kg)	rBM	Tail vein	1 × 10^6^	14 days	3 weeks
Cheng et al. (2017) [29]	Rat, Lewis, M	10	10	MCT (60 mg/kg)	rBM	Tail vein	3 × 10^6^	21 days	3 weeks
Huang et al.(2016) [31]	Rat, SPD, M	6	6	MCT (60 mg/kg)	iP	Tail vein	2 × 10^6^	14 days	4 weeks
Chen et al.(2016) [28]	Rat, SPD, M	8	8	MCT (60 mg/kg)	hUCB	Caudal vein	1 × 10^6^	5 days	3 weeks
Jiang et al.(2012) [32]	Rat, SPD, NR	8	43	MCT (60 mg/kg)	rBM	Tail vein	4 × 10^6^	3 days	3 weeks
Kang et al. (2015) [33]	Rat, SPF, M	10	10	MCT (60 mg/kg)	hUCB	Tail vein	2.5 × 10^5^	14 days	2 weeks
Kimet al (2012) [34]	Rat, SPD, M	6	6	MCT (60 mg/kg)	rBM	Tail vein	2 × 10^7^	7 days	1 week
		6	6						3 weeks
Kim et al. (2016) [35]	Rat, SPD, M	6	6	MCT (60 mg/kg)	hUCB	External jugular vein	3 × 10^6^	7 days	1 week
									3 weeks
		6	6						
		8	8						
Liang et al. (2015) [38]	Rat, SPD, M	10	10	MCT (40 mg/kg)	rADM	Left external jugular vein	1 × 10^6^	7 days	2 weeks
		8	10						3 weeks
Liu et al. (2015) [40]	Rat, SPD, F	8	8	MCT (60 mg/kg)	hUCB	Caudal vein	1 × 10^6^	5 days	3 weeks
Luan et al. (2013) [43]	Rat, SPD, M	10	10	MCT (50 mg/kg)	rBM	Sublingual vein	1 × 10^7^	7 days	23 weeks
Luo et al. (2014) [44]	Rat, SPD, M	8	8	MCT (40 mg/kg)	rADM	Left jugular vein	1 × 10^6^	14 days	1 week
		8	8						2 weeks
		8	8						3 weeks
Raoul et al. (2007) [45]	Mouse, C57BL/6, F	5	5	MCT (5 mg/kg)	rBM	Tail vein	2.5 × 10^6^	3 days	3 weeks
Rathinasabapathy et al. (2016) [46]	Rat, SPD, M	8	8	MCT (50 mg/kg)	rADM	Jugular vein	1 × 10^6^	14 days	2 weeks
Somanna et al. (2014) [47]	Rat, SPD, NR	6	6	MCT (60 mg/kg)	hADM	Intratracheal	3 × 10^6^	14 days	2 weeks
Zhang et al. (2012) [49]	Mouse, ICR, NR	10	10	MCT (400 mg/kg)	rBM	Tail vein	5 × 10^5^	7 days	2 weeks
Zhang et al. (2012) [50]	Rat, SPD, M	9	9	MCT (50 mg/kg)	hUCB	Sublingual vein	3 × 10^6^	7 days	2 weeks
Umar et al. (2009) [48]	Rat, Wistar, F	10	10	MCT (60 mg/kg)	rBM	Jugular vein	1 × 10^6^	14 days	2 weeks
		10	10						3 weeks
		10	10						4 weeks
Liu et al. (2011) [41]	Rat, Wistar, M	17	16	Surgical operation	rADM	Right jugular vein	5 × 10^7^	84 days	2 weeks
Takemiya et al. (2009) [15]	Rat, Lewis, M	10	10	MCT (60 mg/kg)	rBM	Tail vein	5 × 10^5^	14 days	2 weeks
Horimoto et al. (2005) [14]	Rat, SPD, M	10	10	MCT (60 mg/kg)	rBM	Right femoral vein	1 × 10^6^	7 days	2 weeks
Lee et al. (2015) [37]	Rat, SPD, M	8	8	MCT (60 mg/kg)	hUCB	External jugular vein	3 × 10^6^	7 days	1 week
		8	8						2 weeks
		8	8						3 weeks

*PAH* pulmonary arterial hypertension, *SPF* specific-pathogen-free, *SPD* Sprague Dawley, *M* male, *F* female, *NR* not reported, *SCs* stem cells, *hBM* human bone marrow mesenchymal tissue, *hUCB* human umbilical cord blood-derived mesenchymal tissue, *rBM* rat bone marrow mesenchymal tissue, *iP* murine-induced pluripotent stem cells, *BM* bone marrow mesenchymal tissue, *rADM* rat adipose-derived stromal tissue, *ADM* adipose-derived stromal tissue

Follow-up (weeks) indicates the observation time of outcomes after stem cell administration

**Table 2:** Detailed information on outcomes extracted from the included studies

Author (year)	Total *N*, *M*, SD of Animals (treatment/control)						
	Primary outcomes			Secondary outcomes			
	RVSP	mPAP	mRVP	RV/LV+S	RV/BW	WA	WT
	N1, MD1, SD1; N2, MD2, SD2	N1, MD1, SD1; N2, MD2, SD2	N1, MD1, SD1; N2, MD2, SD2	N1, MD1, SD1; N2, MD2, SD2	N1, MD1, SD1; N2, MD2, SD2	N1, MD1, SD1; N2, MD2, SD2	N1, MD1, SD1; N2, MD2, SD2
Lim et al.(2016) [39]	5,34.95,1.57;5,39.79,1.57			5,0.47,0.03;5,0.52,0.01			
Lee et al. (2017) [36]			6,16,1.63;4,29,3.63	6,0.31,0.02;4,0,36,0.0025	4,0.91,0.03;4,0.69,0.01		6,32.52,0.91;4,42.31,0.9
			6,14,0.75;4,49.5,8	6,0.69,0.04;4,0.8,0.08	4,1.75,0.03;4,0.62,0.01		4,34.76,2.71;4,38.22,3.1
			4,14,0.75;4,27,2	4,0.32,0.0025;4,0.4,0.01	4,0.85,0.02;4,0.69,0.01		4,36.16,0.93;4,40.9,2.08
			4,13,1.75;4,44,1.75	4,0.62,0.02;4,0.81,0.06	4,1.52,0.04;4,0.62,0.01		
Guo et al. (2013) [30]	10,94.69,9.03;10,95.55,12.53	10,29.99,6.08;10,30.26,7.83		10,0.34,0.06;10,0.37,0.08			
Luan et al.(2012) [42]	20,43.83,2.13;20,56.84,1.54	20,26.82,2.13;20,41.37,2.24	20,28.34,1.98;20,41.7,6.17		20,0.547,0.04;20,0.613,0.07		20,20.83,5.49;20,45.21,4.37
Baber et al. (2006) [25]		10,33.48,6;10,44.3,6.35		10,0.47,0.03;10,0.6,0.02			
Chen et al. (2014) [27]			7,13.51,1.25;7,29.9,0.71	7,0.34,0.03;7,0.56,0.02		7,78.62,2.58;7,95.17,10.35	7,49.18,3.46;7,73.77,2.77
Chen et al. (2016) [26]	10,28,1.57;10,43,3.18			10,0.55,0.08;10,0.78,0.41			10,21.21,0.02;10,29.16,0.04
Cheng et al. (2017) [29]	10,43.76,2.11;10,60.85,1.2			10,0.43,0.05;10,0.58,0.02	10, 0.42, 0.05;10, 0.51, 0.09		
Huang et al.(2016) [31]	6,47.99,5.53;6,66.7,1.74			6, 0.48, 0.04;6, 0.68, 0.09			
Chen et al.(2016) [28]	8, 29.01,1.67; 8,45.81,2.63						8,18.76,1.09; 8,30.44,2.23
Jiang et al.(2012) [32]	43, 28.84, 1.35; 18, 40.2, 3.46			43, 0.38, 0.05; 18, 0.58, 0.03			43, 36.24, 1.51; 18, 53.47, 3.52
Kang et al. (2015) [33]	10, 32.72, 1.19; 10, 42.24, 7.52			10, 0.5, 0.01; 10, 0.54, 0.07			10.32.4, 4; 10, 42, 7.4
Kimet al (2012) [34]			6, 15.4, 3.8; 6, 38.4, 7.4	6, 0.33, 0.06; 6, 0.53, 0.04	6, 0.73, 0.02; 6, 0.82, 0.05		6, 29.3, 5.2; 6, 38.8, 1.9
			6, 14.8, 3.6; 6, 42.3, 13	6, 0.64, 0.06; 6, 0.79, 0.04	6, 1.47, 0.02; 6, 1.7, 0.09		6, 35.7, 5.7; 6, 40.2, 5.4
Kim et al. (2016) [35]			6, 16.4, 2.1; 6, 34.8, 7.3	6, 0.34, 0.27; 6, 0.38, 0.05	6, 0.75, 0.02; 6, 0.82, 0.05		6, 31.07, 4.71; 6, 38.84, 1.94
			6, 13.4, 2.5; 6, 42.2, 13	6, 0.48, 0.1; 6, 0.79, 0.04	6, 1.3, 0.05; 6, 1.7, 0.07		6, 33.64, 3.57; 6, 40.19, 5.38
Liang et al. (2015) [38]		10, 16.37, 0.94; 10, 26.88, 0.86					
		10, 18.26, 1.41; 8, 32.22, 0.71					
Liu et al. (2015) [40]	8, 29.86, 2.87; 8, 47.13, 3.31						8, 17.53, 1.24; 8, 31.78, 2.16
Luan et al. (2013) [43]		10, 31.57, 5.54; 10, 44.97, 4.26		10, 0.43, 0.05; 10, 0.59, 0.03		10, 45.69, 4.21; 10, 56.52, 4.5	10, 26.28, 3.95; 10, 36.56, 3.68
Luo et al. (2014) [44]		8, 18.63, 2.15; 8, 24.53, 2.9		8, 0.36, 0.04; 17, 0.43, 0.12		8, 58.23, 4.08; 8, 68.7, 3.43	8, 38.06, 4.15; 8, 46.09, 4.7
		8, 23.07, 2.84; 8, 33.18, 3.28		8, 0.39, 0.04; 8, 0.49, 0.02		8, 62.97, 6.58; 8, 80.48, 6.19	8, 40.91, 5.24; 8, 57.26, 4.32
		8, 22.98, 3.24; 8, 36.38, 3.28		8, 0.41, 0.01; 8, 0.51, 0.01		8, 65.27, 5.45; 8, 84.01, 2.76	8, 41.91, 5.16; 8, 64.64, 3.86
Raoul et al. (2007) [45]	5, 17.65, 2.16; 5, 27.77, 0.94			5, 0.3, 0.03; 5, 0.35, 0.02			
Rathinasabapathy et al. (2016) [46]							
Somanna et al. (2014) [47]		6, 52.6, 2.79; 6, 56.24, 3.1	6, 053, 0.04; 6, 0.51, 0.03	6, 0.53, 0.04; 6, 0.51, 0.03			
Zhang et al. (2012) [49]	9, 32.85, 3.44; 9, 59.12, 4.32			9, 0.23, 0.01; 9, 0.31, 0.01			9, 29.97, 2.21; 9, 53.47, 3.52
Zhang et al. (2012) [50]	10, 43.83, 2.13; 10, 56.84, 1.54	10, 26.82, 3.42; 10, 41.37, 2.24	10, 28.34, 1.98; 10, 41.7, 6, 17		10, 0.547, 0.041; 10, 0.613, 0.072		
Umar et al. (2009) [48]	10, 31, 4; 10, 42, 17			10, 0.32, 0.07; 10, 0.47, 0.12	10, 0.64, 0.01; 10, 0.93, 0.21		
Liu et al. (2011) [41]		16, 19.83, 2.32; 17, 33.33, 5.36		16, 0.36, 0.04; 17, 0.43, 0.12			
Takemiya et al. (2009) [15]	10, 43.73, 3.37; 10, 65.56, 2.3						
Horimoto et al. (2005) [14]	10, 42.3, 1.1; 10, 58.7, 1.86				10, 0.462, 0.029; 10, 0.59, 0.084		
Lee et al. (2015) [37]			8, 23.6, 7.6; 8, 36.5, 3.1	8, 1.43, 0.14; 8, 1.47, 0.18	8, 1.14, 0.21; 8, 1.01, 0.1		8, 34.2, 4; 8, 38.5; 4
			8, 21.3, 4.9; 8, 37.2, 6.3	8, 2.18, 0.45; 8, 2.89, 0.43	8, 1.72, 0.33; 8, 1.62, 0.24		8, 33.2, 3.9; 8, 38.8, 6.3
			8, 16.5, 3.6; 8, 52, 19.7	8, 1.85, 0.28; 8, 2.88, 0.58	8, 1.81, 0.15; 8, 2.67, 0.04		8, 37.3, 1.5; 8, 32.3; 2.5

*N* sample number, *M* mean, *SD* standard deviation, *RVSP* right ventricular systolic pressure, *mPAP* mean pulmonary arterial pressure, *mRVP* mean right ventricle pressure, *RV/LV+S* the weight ratio of the right ventricle to the left ventricle plus septum, *RV/BW* right ventricle to body weight ratio, *WA* pulmonary arteriole area index, *WT* wall thickness of pulmonary arteriole

### Risk of bias

All 28 articles that met the inclusion criteria for this meta-analysis were included (Table 3). None of the experiments were judged as having a low risk of bias across all domains. All studies reported similar experimental and control groups at baseline, which reduced the risk of selection bias based on animal characteristics. Although the assignment of subjects to the experimental and control groups was random, none of the studies clearly described the method of random sequence generation. For this reason, the risk of bias in the sequence generation domain was judged as “unclear” in all studies. However, although none of the studies adequately described the method used to conceal allocation, the animals were randomly housed, the caregivers and investigators were blinded to the intervention each animal received, random outcome assessment was reported, and blinding of the outcome assessor was documented. Using the signalling questions provided, all studies were scored as having a low risk of attrition and reporting bias. Furthermore, we did not identify any additional sources of bias that were not already covered by the SYRCLE Risk of Bias tool.

**Table 3:** SYRCLE risk of bias assessment for the included studies

Author (year)	Random sequence generation?	Groups similar at baseline?	Allocation concealed?	Animals randomly housed?	Blinding of caregivers and/or examiners?	Random selection for outcome assessment?	Blinding of outcome assessor?	Incomplete outcome data addressed?	Free from selective outcome reporting?	Free from other bias?
Lim et al.(2016) [39]	U	L	U	U	U	U	U	L	L	L
Lee et al. (2017) [36]	U	L	U	U	U	U	H	L	L	L
Guo et al.(2013) [30]	U	L	U	U	U	U	U	L	L	L
Luan et al. (2012) [42]	U	L	U	U	U	U	U	L	L	L
Baber et al. (2006) [25]	U	L	U	U	U	U	U	L	L	L
Chen et al. (2014) [27]	U	L	U	U	U	U	U	L	L	L
Chen et al. (2016) [28]	U	L	U	U	U	U	U	L	L	L
Cheng et al. (2017) [29]	U	L	U	U	U	U	U	L	L	L
Huang et al. (2016) [31]	U	L	U	U	U	U	U	L	L	L
Chen et al. (2016) [26]	U	L	U	U	U	U	U	L	L	L
Jiang et al. (2012) [32]	U	L	U	U	U	U	U	L	L	L
Kang et al. (2015) [33]	U	L	U	U	U	U	U	L	L	L
Lee et al. (2015) [37]	U	L	U	U	U	U	U	L	L	L
Kim et al. (2012) [34]	U	L	U	U	U	U	U	L	L	L
Kim et al. (2016) [35]	U	L	U	U	U	U	U	L	L	L
Liang et al. (2015) [38]	U	L	U	U	U	U	U	L	L	L
Liu et al. (2015) [40]	U	L	U	U	U	U	U	L	L	L
Luan et al. (2013) [43]	U	L	U	U	U	U	U	L	L	L
Luo et al. (2014) [44]	U	L	U	U	U	U	U	L	L	L
Raoul et al. (2007) [45]	U	L	U	U	U	U	U	L	L	L
Rathinasabapathy et al. (2016) [46]	U	L	U	U	U	U	U	L	L	L
Somanna et al. (2014) [47]	U	L	U	U	U	U	U	L	L	L
Takemiya et al. (2009) [15]	U	L	U	U	U	U	U	L	L	L
Zhang et al. (2012) [49]	U	L	U	U	U	U	U	L	L	L
Zhang et al. (2012) [50]	U	L	U	U	U	U	U	L	L	L
Liu et al. (2011) [41]	U	L	U	U	U	U	U	L	L	L
Umar et al. (2009) [48]	U	L	U	U	U	U	U	L	L	L
Kanki et al.(2005) [14]	U	L	U	U	U	U	U	L	L	L

*H* high risk of bias, *L* low risk of bias, *U* unclear risk of bias

### Effect of SC therapy on PAH

**Fig. 2:**
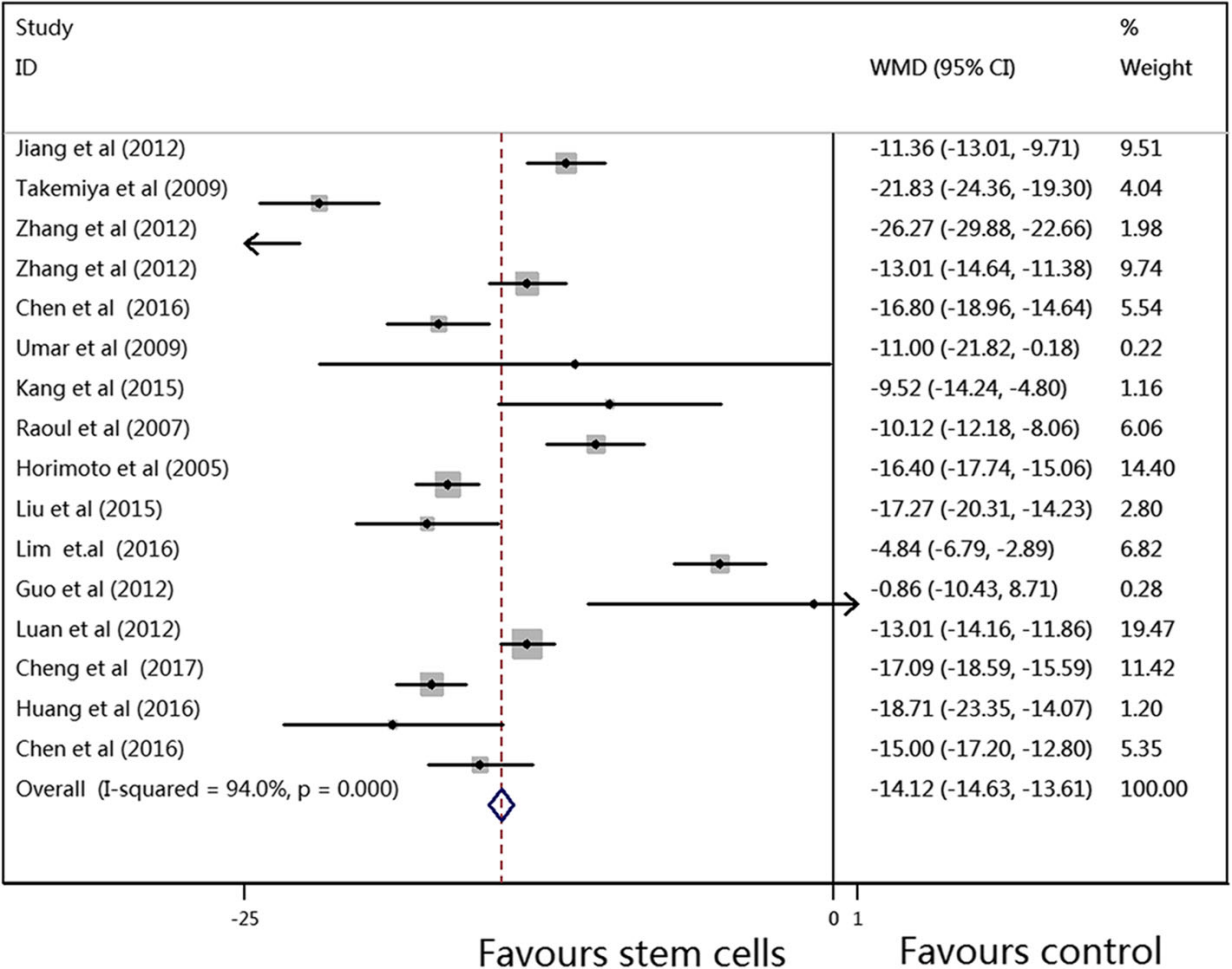
Meta-analysis of overall pooled WMDs with 95% CIs across studies for primary outcomes in PAH. Forest plot showing that SC therapy significantly reduced the RVSP in animals with PAH from a random-effects model. Abbreviations: WMD, weighted mean difference; RVSP, right ventricular systolic pressure; PAH, pulmonary arterial hypertension

**Fig. 3:**
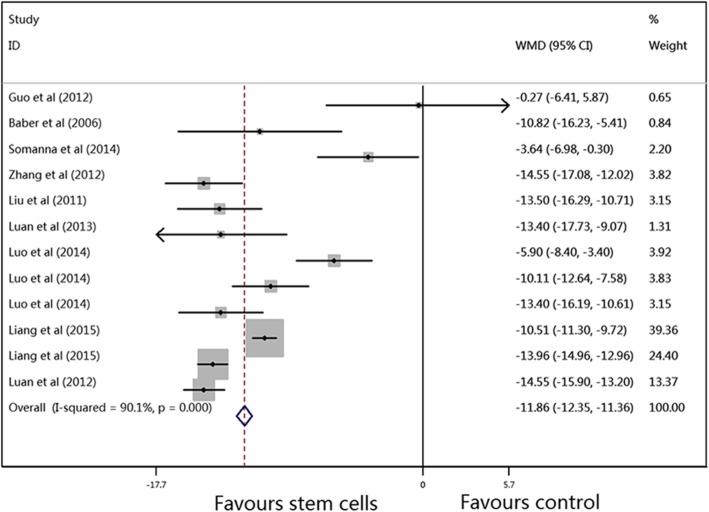
Meta-analysis of overall pooled WMDs with 95% CIs across studies for primary outcomes in PAH. Forest plot showing that SC therapy significantly reduced the mPAP in animals with PAH from a random-effects model. Abbreviations: WMD, weighted mean difference; mPAP, mean pulmonary arterial pressure; PAH, pulmonary arterial hypertension

**Fig. 4:**
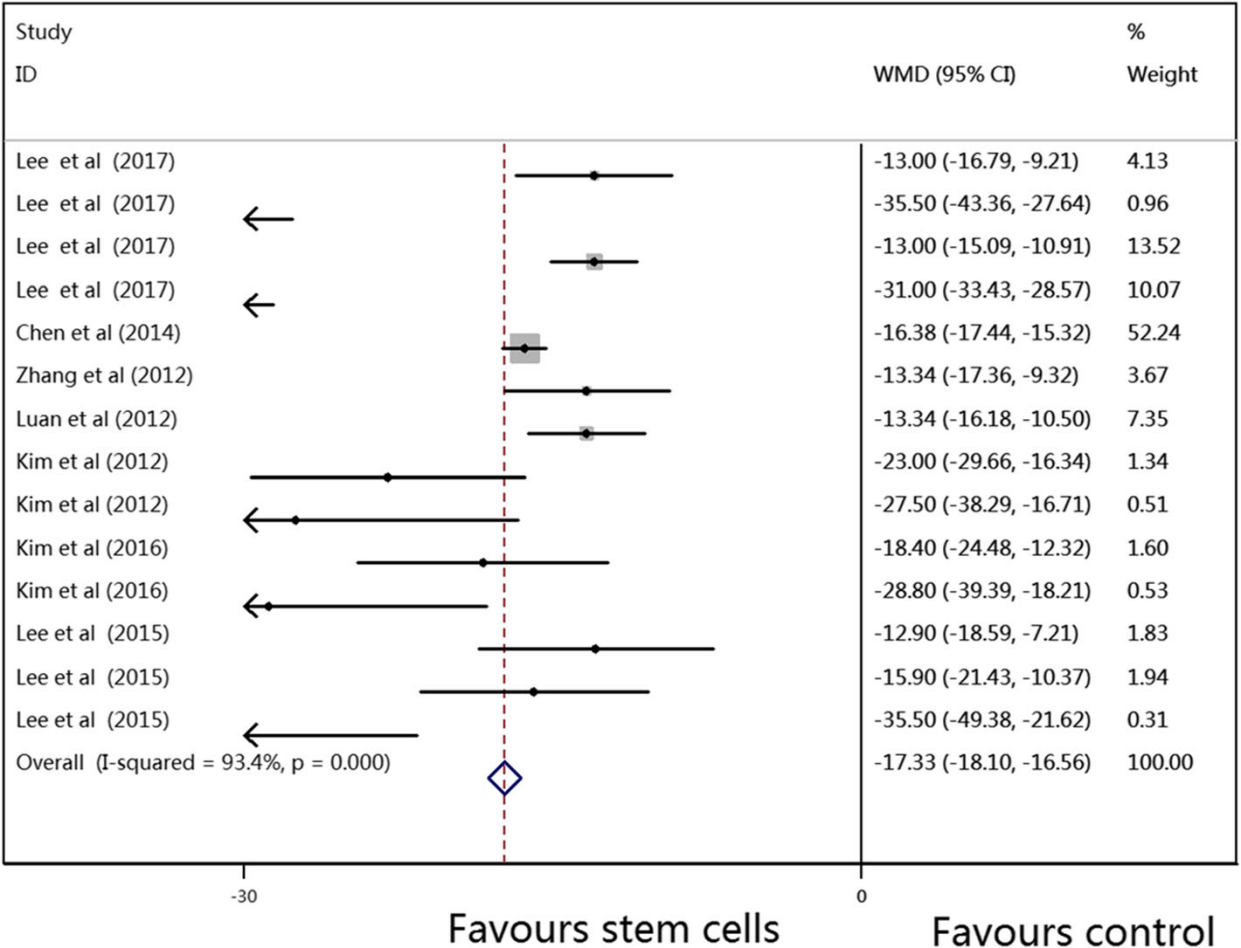
Meta-analysis of overall pooled WMDs with 95% CIs across studies for primary outcomes in PAH. Forest plot showing that SC therapy significantly reduced the mRVP in animals with PAH from a random-effects model. Abbreviations: WMD, weighted mean difference; mRVP, mean right ventricle pressure; PAH, pulmonary arterial hypertension

The results of the primary outcomes in this meta-analysis are shown in Figs. 2, 3 and 4. The pooled WMDs (95% CIs) for RVSP, mPAP, and mRVP were 14.12 (− 14.63, − 13.61), − 11.86 (− 12.35, − 11.36), and − 17.33 (− 18.10, − 16.56), and the *P* values were all < 0.001, which indicated that compared to vehicle treatment, SC therapy was significantly associated with reduced RVSP, mPAP, and mRVP values in the animal model of PAH. A random-effects model was used to perform this meta-analysis, as there was significant heterogeneity (*I*^2^ = 94.0%, 90.1%, and 93.4%, respectively) among the studies.

The results of secondary outcomes in this meta-analysis are shown in Additional file 1: Figure S1, Additional file 2: Figure S2, Additional file 3: Figure S3, and Additional file 4: Figure S4. The pooled WMDs (95% CIs) for RV/LV+S, RV/BW, WA, and WT were 0.10 (− 0.10, − 0.09), 0.23 (0.21, 0.24), − 13.66 (− 15.71, − 11.62), and − 7.96 (− 7.99, − 7.93), respectively, and the *P* values were all < 0.001, which indicated that compared to vehicle treatment, SC therapy was also significantly associated with reduced RV/LV+S, RV/BW, WA, and WT values in the animal model of PAH. A random-effects model was also used to perform this meta-analysis, as there was moderate or significant heterogeneity (*I*^2^ = 93.2%, 99.8%, 67.9%, and 97.5%, respectively) among the studies. In summary, based on the results of forest plots of the primary and secondary outcomes, we concluded that SC therapy is effective for PAH in animal studies.

### Stratified analysis and meta-regression analysis

Stratified analysis was conducted on the primary outcomes according to source, route, dose, and timing of SC treatment after PAH and follow-up. However, we could not find the source of heterogeneity. To further investigate the unexplained heterogeneity across these studies, meta-regression was performed to simultaneously examine the impact of all variables on the study effect. However, for RVSP, no significant sources of heterogeneity were found. For mPAP, the method of establishing the PAH model and the dose of MCT were the sources of heterogeneity (*P* = 0.046). For mRVP, the follow-up time was the significant source of heterogeneity (*P* = 0.007).

### Publication bias

Funnel plots and Egger’s linear regression tests were performed to evaluate publication bias in RVSP, mPAP, mRVP, RV/LV+S, RV/BW, and WT individually (Additional file 5: Figure S5, Additional file 6: Figure S6, Additional file 7: Figure S7, Additional file 8: Figure S8, Additional file 9: Figure S9, and Additional file 10: Figure S10). As the number of included studies that measured WA was small (< 10), we did not construct a funnel plot, as it may not have detected publication bias [51]. No significant publication bias was found for mPAP (Begg’s test, *P* = 0.244; Egger’s test, *P* = 0.423; Additional file 6: Figure S6), mRVP (Begg’s test, *P* = 0.003; Egger’s test, *P* = 0.335; Additional file 7: Figure S7), RV/LV+S (Begg’s test, *P* = 0.129; Egger’s test, *P* = 0.155; Additional file 8: Figure S8), RV/BW (Begg’s test, *P* = 0.752; Egger’s test, *P* = 0.186; Additional file 9: Figure S9), or WT (Begg’s test, *P* = 0.492; Egger’s test, *P* = 0.050; Additional file 10: Figure S10) in PAH. However, significant publication bias was found for RVSP (Begg’s test, *P* = 0.013; Egger’s test, *P* = 0.000; Additional file 5: Figure S5).

## Discussion

Systematic reviews play a critical role in applying preclinical data to clinical practice. When combined with a meta-analysis of these experiments, the results can be assessed in a more methodical and objective manner. In preclinical studies of PAH animal models, SCs have been shown to improve pulmonary pressure, RV hypertrophy, and pulmonary artery endothelium over-proliferation [52]. Investigators using animal models of PAH have reported similarly promising results. Progress in regenerative medicine has led to the first clinical trial to evaluate the safety of autologous endothelial progenitor cells in PAH [53], but to date, SCs have not been used for clinical treatment in patients with PAH. To our knowledge, this is the first attempt to systematically collect and evaluate the current preclinical evidence supporting the use of SCs in animal models of PAH. Based on the results of this meta-analysis, we prove that SCs do indeed have potential therapeutic efficacy for reducing pulmonary artery pressure and RV remodelling in PAH animal models. Furthermore, these results are applicable across a range of experimental conditions.

SCs significantly improved PAH and RV pressure. Our results showed that SCs could ameliorate pulmonary artery resistance and inhibit the over-proliferation of pulmonary epithelial cells, which is consistent with studies that used SCs to treat adult animal models of lung diseases. For instance, a meta-analysis performed by Zhao et al. [54] found a positive effect on pulmonary artery resistance in lung disease. In our study, the mPAP results yielded a WMD of − 11.86, which is comparable to the values obtained in the majority of studies but is different from the values obtained by Guo et al. [30]. Taken together, these findings support the potential use of SC therapy in preclinical studies of PAH.

This meta-analysis suggests that further studies should be performed to elucidate the ideal MSC dose, as the outcomes for mRVP suggested doses between 0.5 ×  10^6^ and 1 × 10^6^ SCs, while the outcomes for mPAP and RVSP showed optimal doses between1 × 10^6^ and 3 × 10^6^. Similarly, there was a discrepancy regarding the most effective route of delivery, with the mPAP and RVSP results favouring the sublingual vein route, while the mRVP studies favoured external jugular vein injection. These variables are exceedingly relevant to future patient applications from a clinical perspective. As such, our findings should be used to guide future preclinical or clinical trials when determining the optimal SC characteristics for successful outcomes.

In all comparisons, significant heterogeneity in treatment effects was found among studies. This heterogeneity can be expected in studies such as ours that are based on a limited number of included studies and have potential bias in study selection. Funnel plots and Egger’s test reported the outcomes of RVSP and confirmed the presence of publication bias. Study quality may also be affected if our primary outcome measure was not the focus of the preclinical study. Such variations in study design could account for the heterogeneity found in this meta-analysis.

We performed a meta-regression analysis to assess the impact of these variables and consider sources of heterogeneity. The results of this analysis suggest that the mPAP, mRVP, and RVSP outcomes were indeed associated with moderator variables in the included study. However, it is also important to consider the limitations of meta-regression. In this meta-analysis, there are relatively few studies but many possible study characteristics that could explain the heterogeneity. Without significant power, it is possible to arrive at false-positive conclusions. Meta-regression is intended to generate hypotheses regarding heterogeneity rather than fully explain heterogeneity. For this reason, it is difficult to truly ascertain the variables with the most promising effects given the current collection of studies.

The SYRCLE Risk of Bias tool highlighted notable deficiencies in reporting across all studies. None of the 28 articles included in this meta-analysis were considered to have a low risk of bias based on the reporting domains included in this tool. As discussed, domains were scored as having a low risk of bias only if the authors specifically stated these details in their published manuscripts. Therefore, it is possible that the studies utilised such methods in their studies but simply failed to report them. This meta-analysis emphasises this widespread shortcoming and suggests a need for higher reporting standards when publishing, specifically for preclinical translational studies. We suggest using a checklist such as the SYRCLE Risk of Bias tool when designing future preclinical studies to minimise internal reporting bias.

The strengths of this meta-analysis are obvious. First, this is the first meta-analysis of the effect of SCs on PAH in animal models. Second, we conducted a systematic literature search and followed a published protocol to ensure a diligent and rigorous review process. Third, data from studies including large samples were pooled in this meta-analysis, increasing its robustness. Furthermore, our primary outcomes of pulmonary artery and RV pressure are widely applicable to future preclinical and clinical trials.

In addition, this meta-analysis has several limitations. For example, the included studies are limited only to studies that have already been published. Unpublished data may exist that would alter our results. While we have made every effort to thoroughly search the current literature, it is possible that we may have missed relevant studies. Additionally, this meta-analysis is limited by relatively small data sets due to strict inclusion criteria, with external publication bias across these studies. Our study did not include studies that used microvesiclesor medium-derived SCs. Finally, we are unable to comment on the clinical safety of SC therapy, as none of the included studies thoroughly investigated SC dose-effects on PAH in animal models. While immunogenicity is less of a concern with SC therapy, other significant risks exist. For instance, SCs, especially induced pluripotent stem cells (iPSCs), have been associated with malignant transformation, tumour growth, and a higher overall degree of metastasis [55–57]. Therefore, iPSCs should be considered carefully for the future treatment of PAH, although none of the studies indicated that iPSCs promoted tumourigenicity in PAH. Although complications have been observed in humans receiving SCs, meta-analysis has not shown a direct correlation between SCs and acute toxicity, systemic failure, malignancy, or death [58–60]. Although there have been clinical trials on the safety and efficacy of SCs for acute respiratory distress syndrome and other respiratory diseases, as well as progenitor cells for pulmonary hypertension, SCs have not yet been used to treat pulmonary hypertension. Therefore, further research is needed to define the dose of SCs for standardised preclinical studies or clinical trials. Despite these limitations, our results reflect the widespread tendencies in this field of research.

## Conclusions

These findings highlight the effects of SCs on pulmonary artery and RV stress and pulmonary artery and RV remodelling in animal models. Furthermore, these results may help to standardise preclinical animal studies and provide a theoretical basis for future SC clinical trial designs.

## Additional files

Additional file 1: **Figure S1.** Meta-analysis of overall pooled WMDs with 95% CIs across studies for secondary outcomes in PAH. Forest plot showing that SC therapy significantly reduced the RV/LV+S in animals with PAH from a random-effects model. Abbreviations: PAH, pulmonary arterial hypertension; RV/LV+S, the weight ratio of the right ventricle to the left ventricle plus septum; WMD, weighted mean difference. (TIF 1900 kb)

Additional file 2: **Figure S2.** Meta-analysis of overall pooled WMDs with 95% CIs across studies for secondary outcomes in PAH. Forest plot showing that SC therapy significantly reduced the RV/BW in animals with PAH from a random-effects model. Abbreviations: PAH, pulmonary arterial hypertension; RV/BW, right ventricle to body weight ratio; WMD, weighted mean difference. (TIF 987 kb)

Additional file 3: **Figure S3.** Meta-analysis of overall pooled WMDs with 95% CIs across studies for secondary outcomes in PAH. Forest plot showing that SC therapy significantly reduced the WA in animals with PAH from a random-effects model. Abbreviations: PAH, pulmonary arterial hypertension; WA, pulmonary arteriole area index; WMD, weighted mean difference. (TIF 358 kb)

Additional file 4: **Figure S4.** Meta-analysis of overall pooled WMDs with 95% CIs across studies for secondary outcomes in PAH. Forest plot showing that SC therapy significantly reduced the WT in animals with PAH from a random-effects model. Abbreviations: PAH, pulmonary arterial hypertension; WT, wall thickness; WMD, weighted mean difference. (TIF 1560 kb)

Additional file 5: **Figure S5.** Funnel plot indicates significant publication bias regarding mPAP in animal studies of PAH. (TIF 347 kb)

Additional file 6: **Figure S6.** Funnel plot indicates no significant publication bias regarding mPAP in animal studies of PAH. (TIF 356 kb)

Additional file 7: **Figure S7.** Funnel plots demonstrating no significant publication bias among the included studies for mRVP in PAH. (TIF 347 kb)

Additional file 8: **Figure S8.** Funnel plots demonstrating no significant publication bias among the included studies for RV/LV+S in PAH. (TIF 357 kb)

Additional file 9: **Figure S9.** Funnel plots demonstrating no significant publication bias among the included studies for RV/BW in PAH. (TIF 340 kb)

Additional file 10: **Figure S10.** Funnel plots demonstrating no significant publication bias among the included studies for WT in PAH. (TIF 360 kb)

Additional file 11: **Table S1.** The PRISMA 2009 checklist. (DOCX 26.7 kb)

Additional file 12: **Table S2.** The detailed search strategy. (DOCX 17.6 kb)

## Abbreviations

mPAP: Mean pulmonary arterial pressure
mRVP: Mean right ventricle pressure
PAH: pulmonary arterial hypertension
RV/BW: Right ventricle to body weight ratio
RV/LV+S: The weight ratio of the right ventricle to the left ventricle plus septum
RVSP: Right ventricular systolic pressure
SCs: Stem cells
SYRCLE: SYstematic Review Centre for Laboratory animal Experimentation
WA: Pulmonary arteriole area index
WMD: Weighted mean difference
WT: Wall thickness of pulmonary arteriole
