# Supplementary figures and images for: Efficacy of stem cell therapy for pulmonary arterial hypertension: a systematic review and meta-analysis of preclinical studies
Source: Stem Cell Res Ther. 2019 Feb 13;10:55. doi: 10.1186/s13287-019-1162-8 (PMC6374914; doi:10.1186/s13287-019-1162-8)

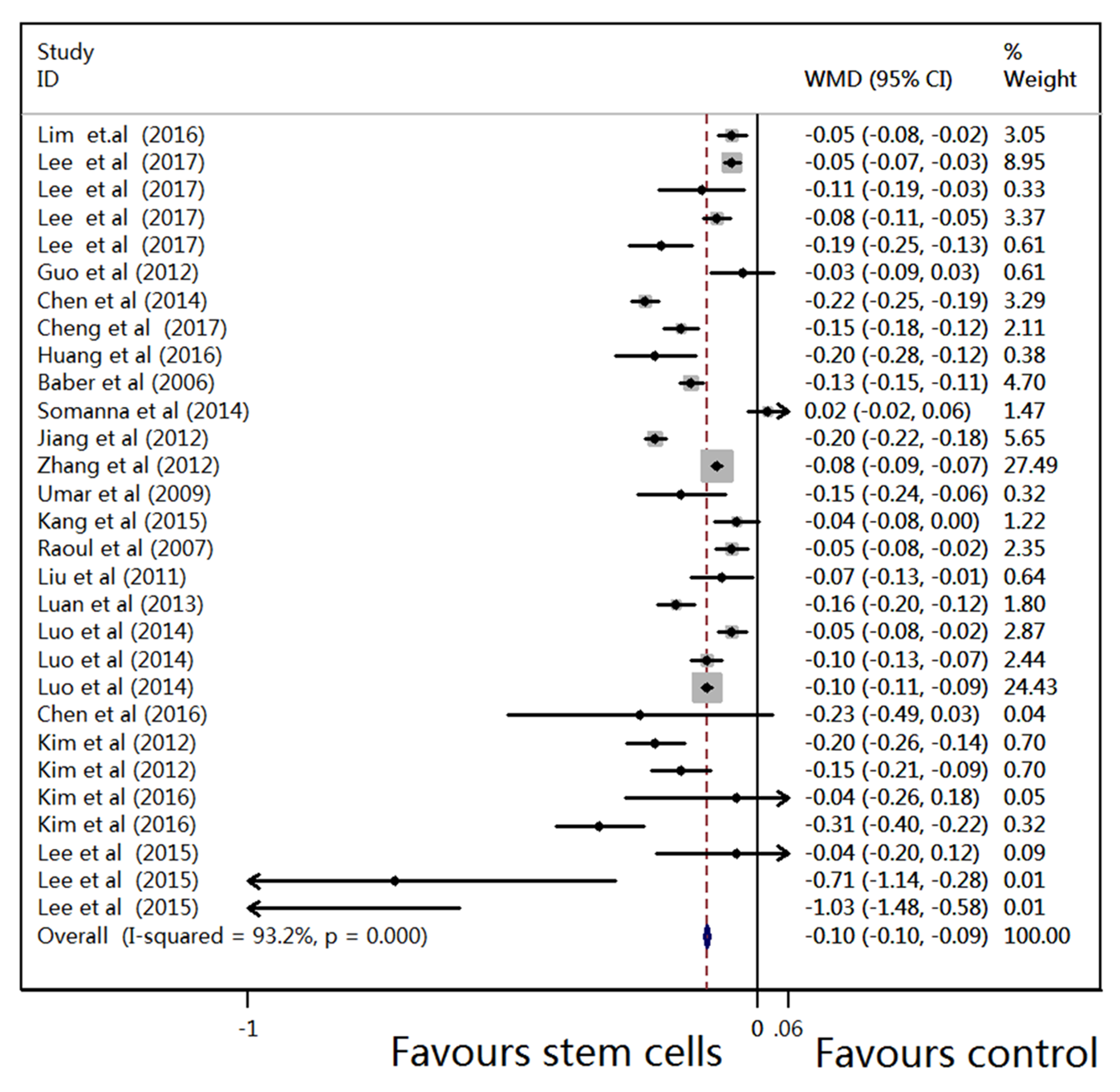

Supplement: Supplementary file 1 — Figure S1. Meta-analysis of overall pooled WMDs with 95% CIs across studies for secondary outcomes in PAH. Forest plot showing that SC therapy significantly reduced the RV/LV+S in animals with PAH from a random-effects model. Abbreviations: PAH, pulmonary arterial hypertension; RV/LV+S, the weight ratio of the right ventricle to the left ventricle plus septum; WMD, weighted mean difference. (TIF 1900 kb) [file 13287_2019_1162_MOESM1_ESM.tif]

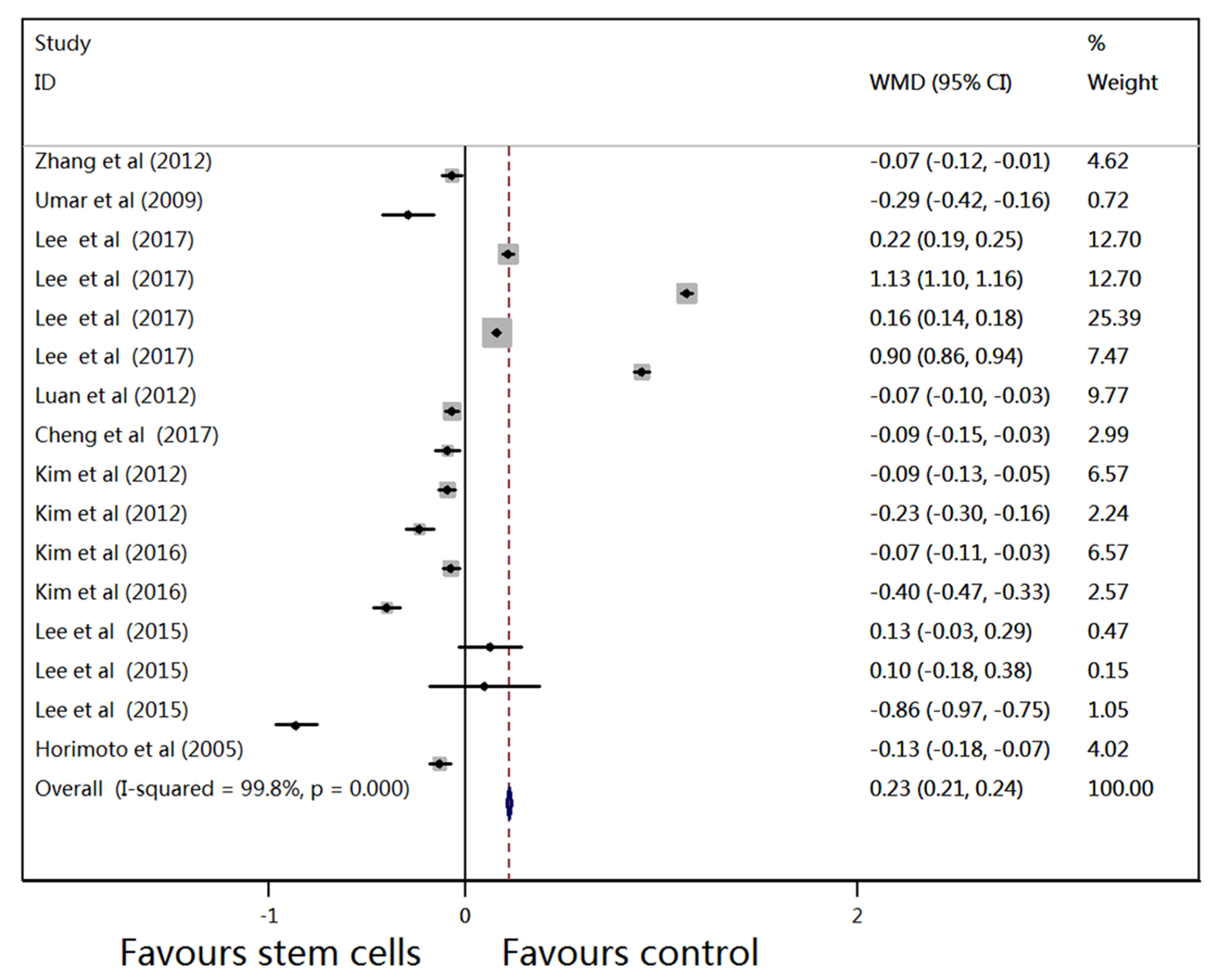

Supplement: Supplementary file 2 — Figure S2. Meta-analysis of overall pooled WMDs with 95% CIs across studies for secondary outcomes in PAH. Forest plot showing that SC therapy significantly reduced the RV/BW in animals with PAH from a random-effects model. Abbreviations: PAH, pulmonary arterial hypertension; RV/BW, right ventricle to body weight ratio; WMD, weighted mean difference. (TIF 987 kb) [file 13287_2019_1162_MOESM2_ESM.tif]

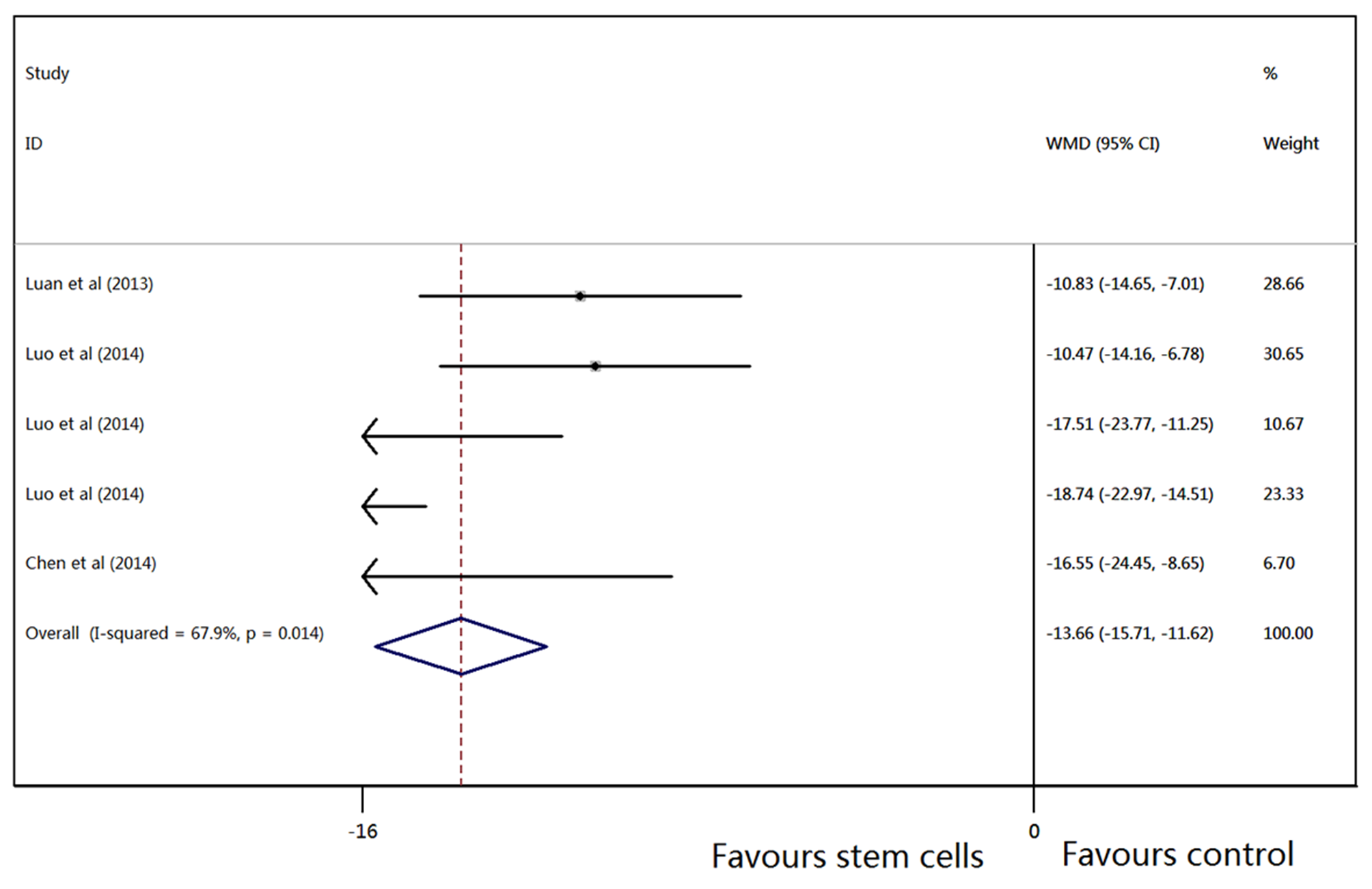

Supplement: Supplementary file 3 — Figure S3. Meta-analysis of overall pooled WMDs with 95% CIs across studies for secondary outcomes in PAH. Forest plot showing that SC therapy significantly reduced the WA in animals with PAH from a random-effects model. Abbreviations: PAH, pulmonary arterial hypertension; WA, pulmonary arteriole area index; WMD, weighted mean difference. (TIF 358 kb) [file 13287_2019_1162_MOESM3_ESM.tif]

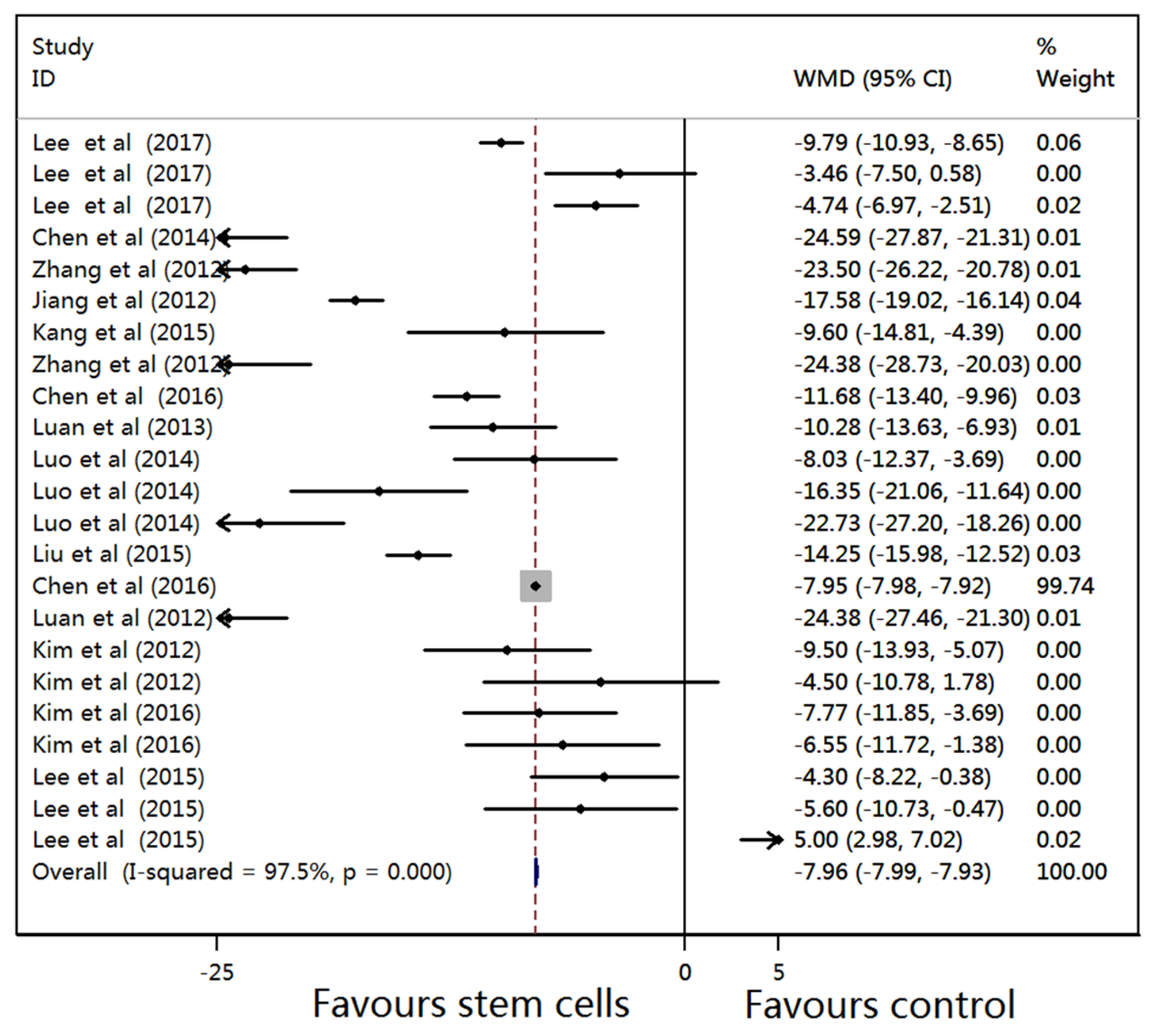

Supplement: Supplementary file 4 — Figure S4. Meta-analysis of overall pooled WMDs with 95% CIs across studies for secondary outcomes in PAH. Forest plot showing that SC therapy significantly reduced the WT in animals with PAH from a random-effects model. Abbreviations: PAH, pulmonary arterial hypertension; WT, wall thickness; WMD, weighted mean difference. (TIF 1560 kb) [file 13287_2019_1162_MOESM4_ESM.tif]

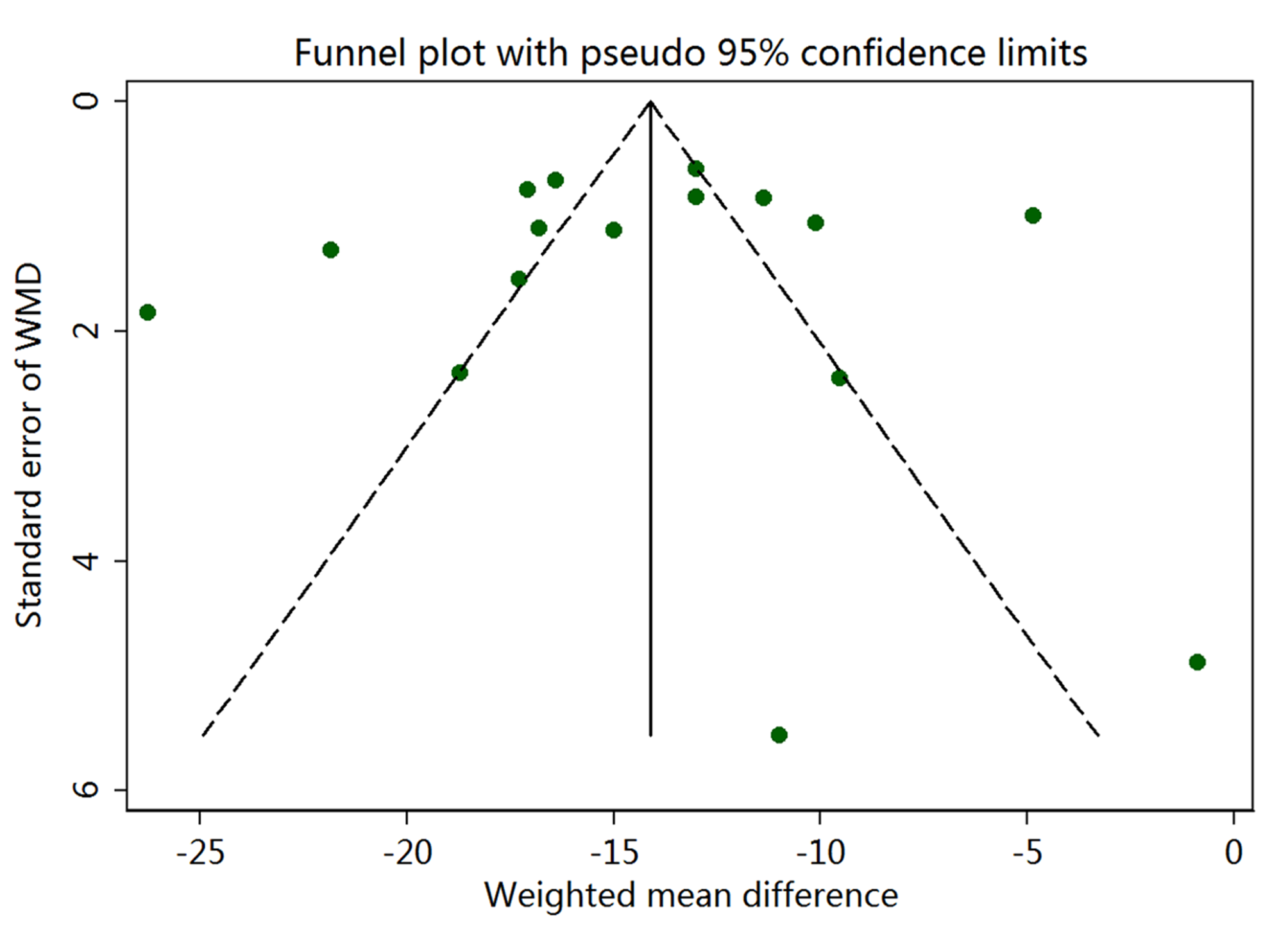

Supplement: Supplementary file 5 — Figure S5. Funnel plot indicates significant publication bias regarding mPAP in animal studies of PAH. (TIF 347 kb) [file 13287_2019_1162_MOESM5_ESM.tif]

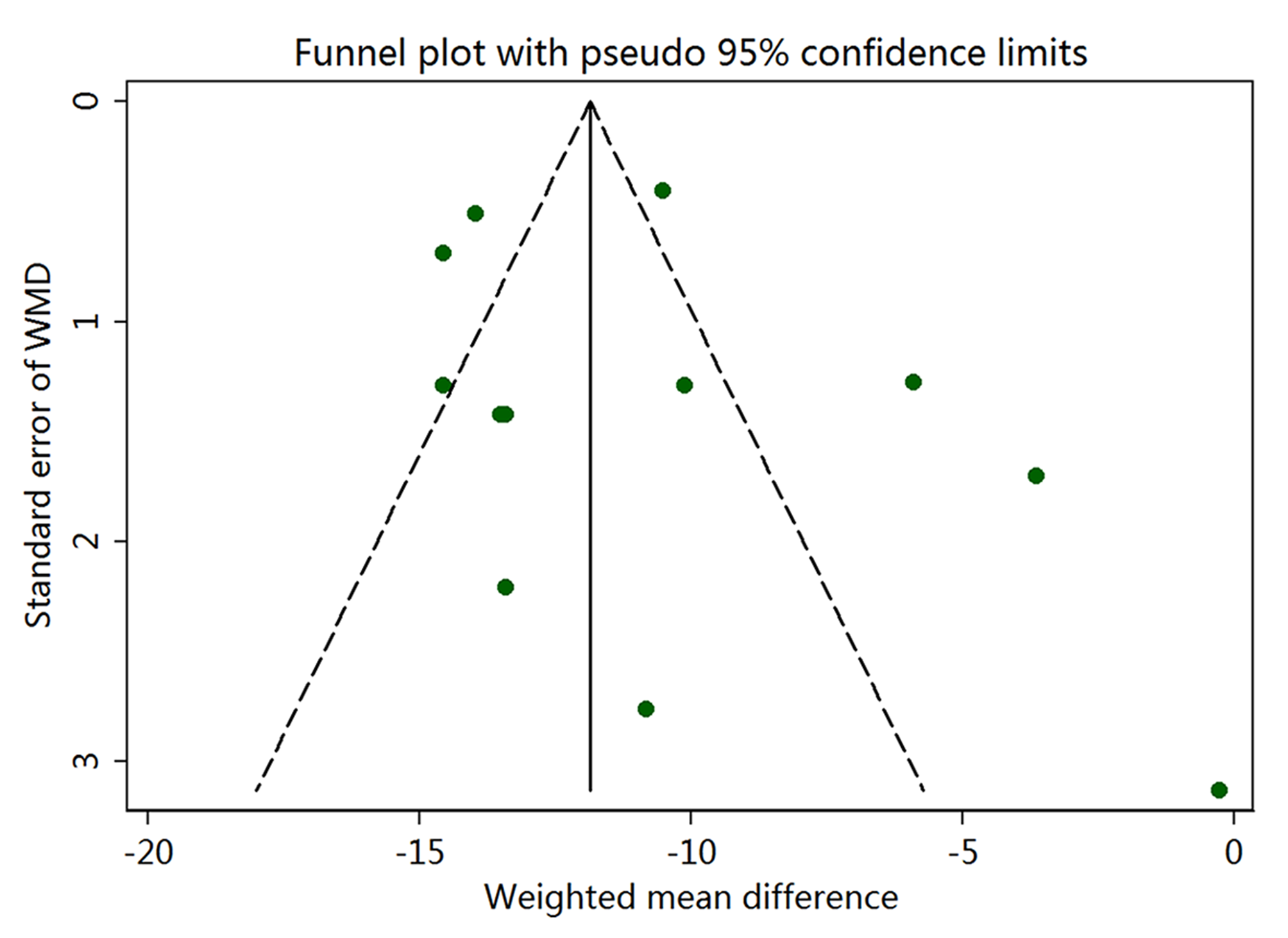

Supplement: Supplementary file 6 — Figure S6. Funnel plot indicates no significant publication bias regarding mPAP in animal studies of PAH. (TIF 356 kb) [file 13287_2019_1162_MOESM6_ESM.tif]

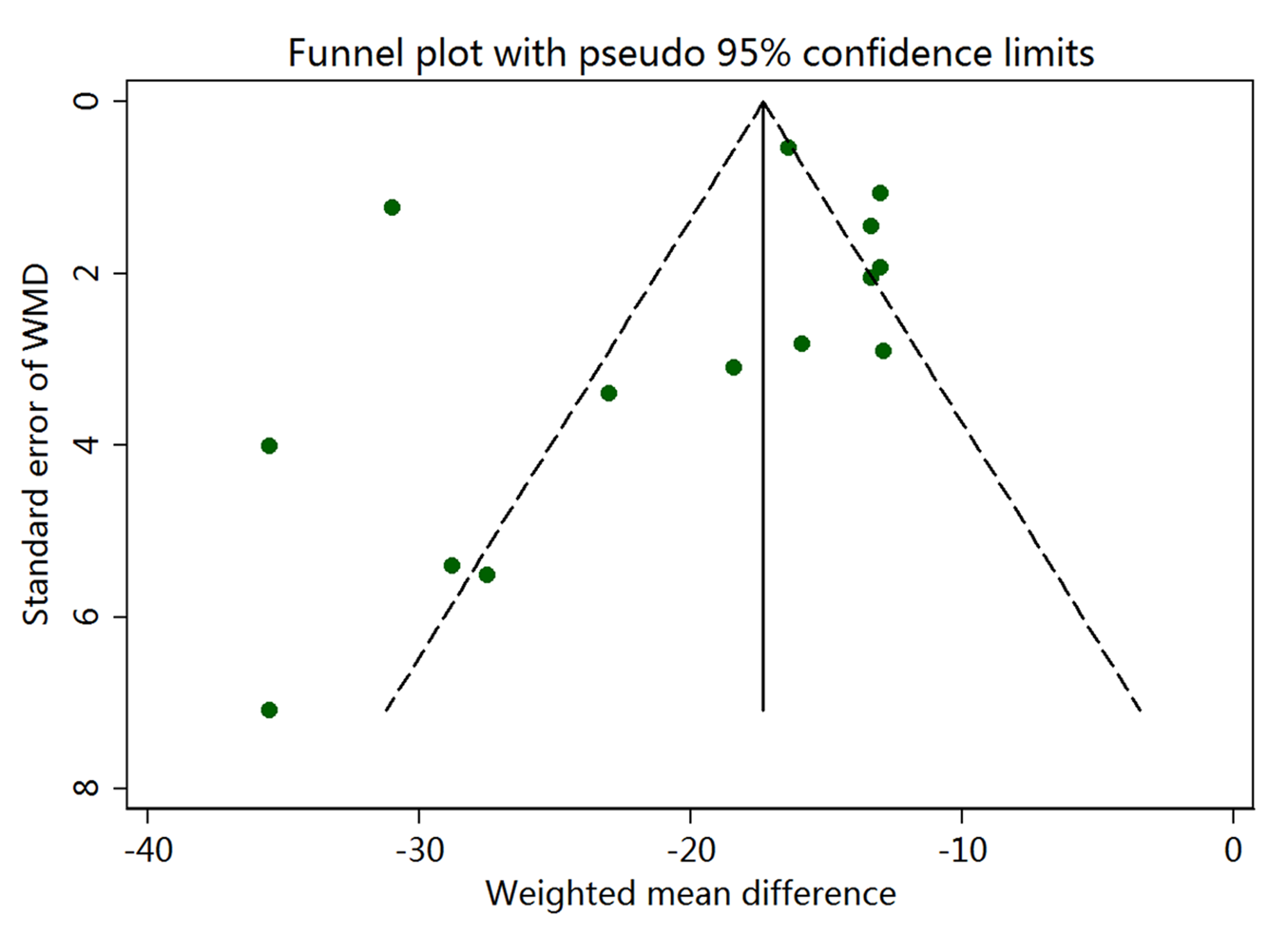

Supplement: Supplementary file 7 — Figure S7. Funnel plots demonstrating no significant publication bias among the included studies for mRVP in PAH. (TIF 347 kb) [file 13287_2019_1162_MOESM7_ESM.tif]

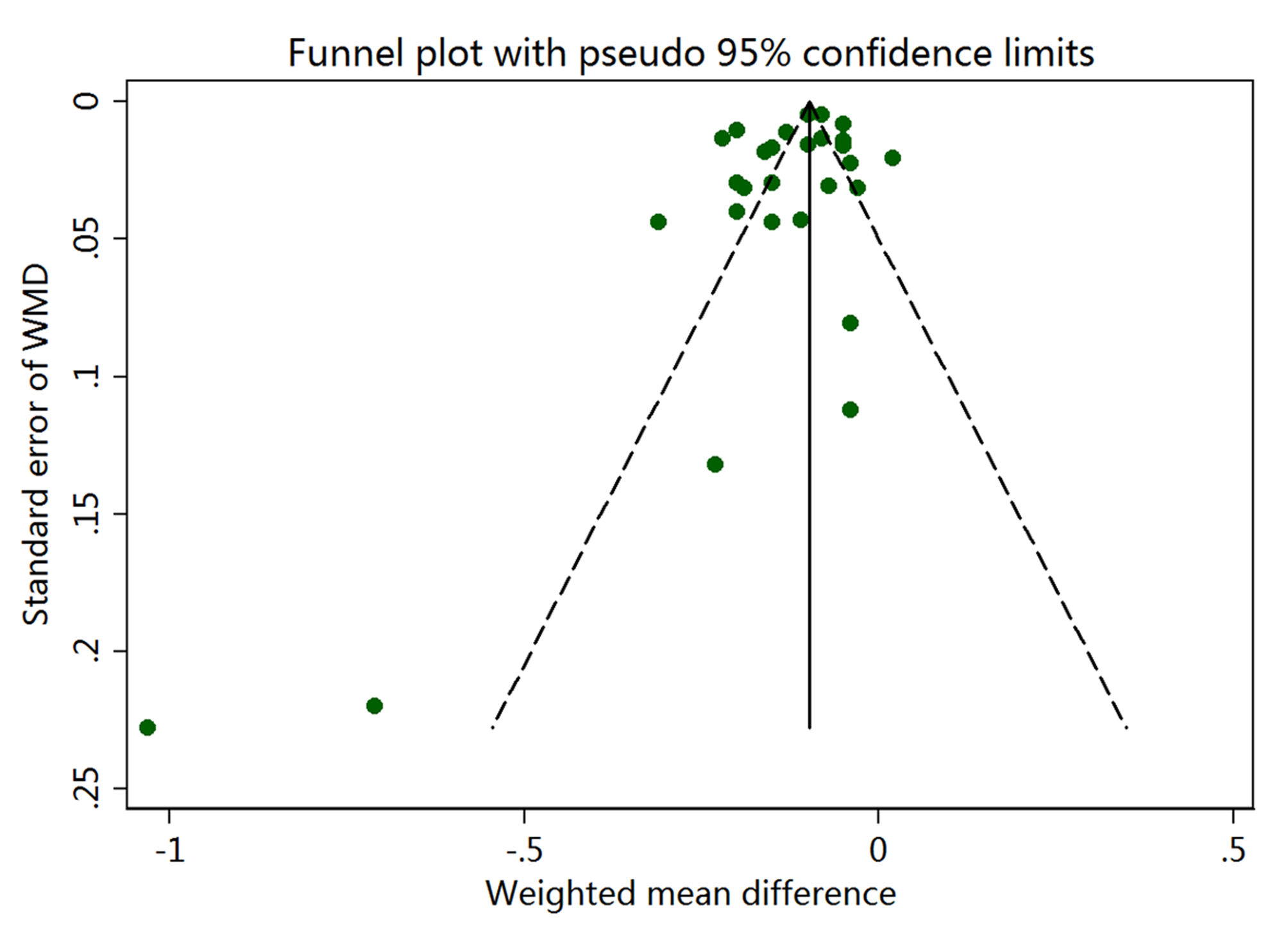

Supplement: Supplementary file 8 — Figure S8. Funnel plots demonstrating no significant publication bias among the included studies for RV/LV+S in PAH. (TIF 357 kb) [file 13287_2019_1162_MOESM8_ESM.tif]

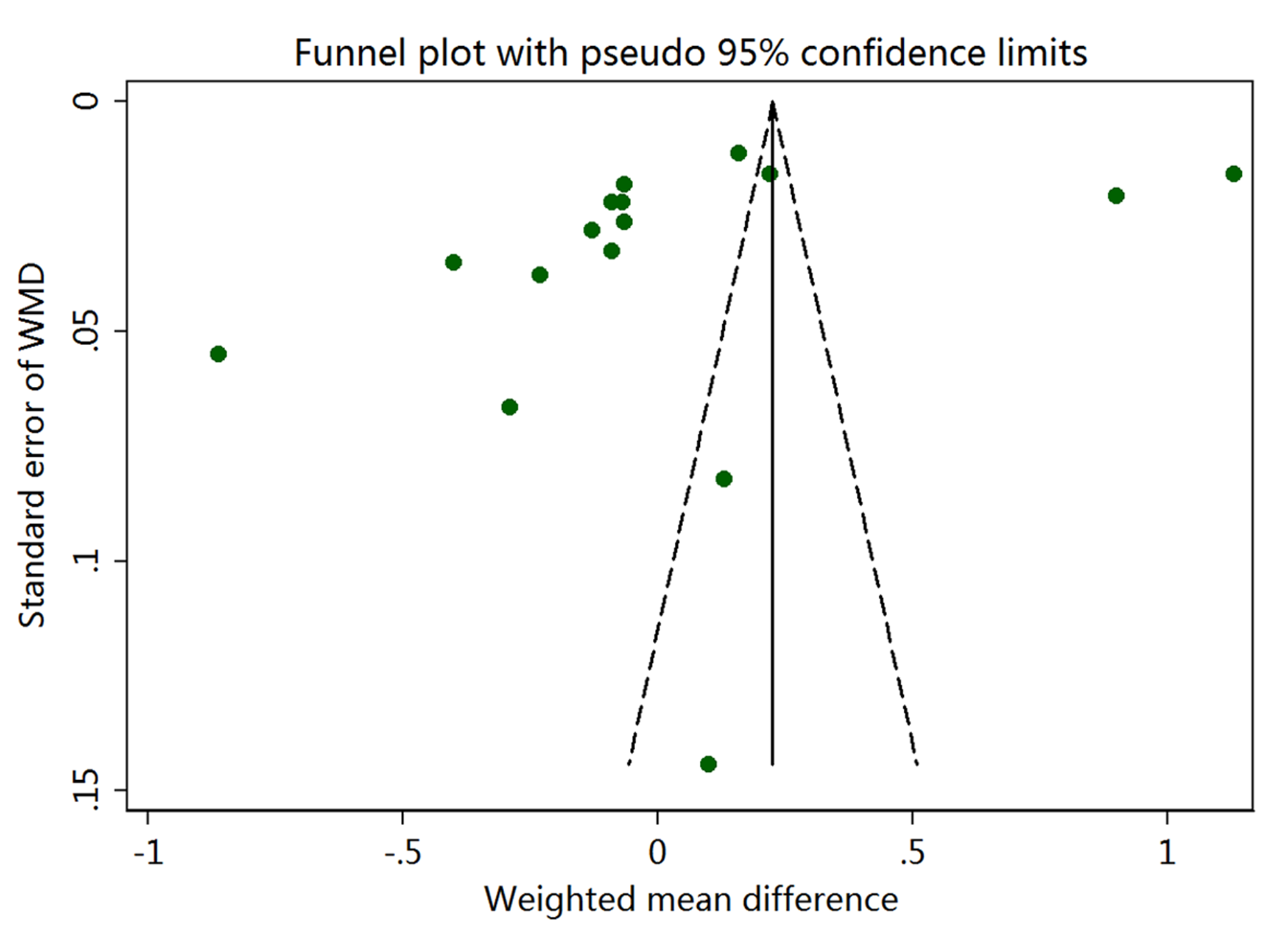

Supplement: Supplementary file 9 — Figure S9. Funnel plots demonstrating no significant publication bias among the included studies for RV/BW in PAH. (TIF 340 kb) [file 13287_2019_1162_MOESM9_ESM.tif]

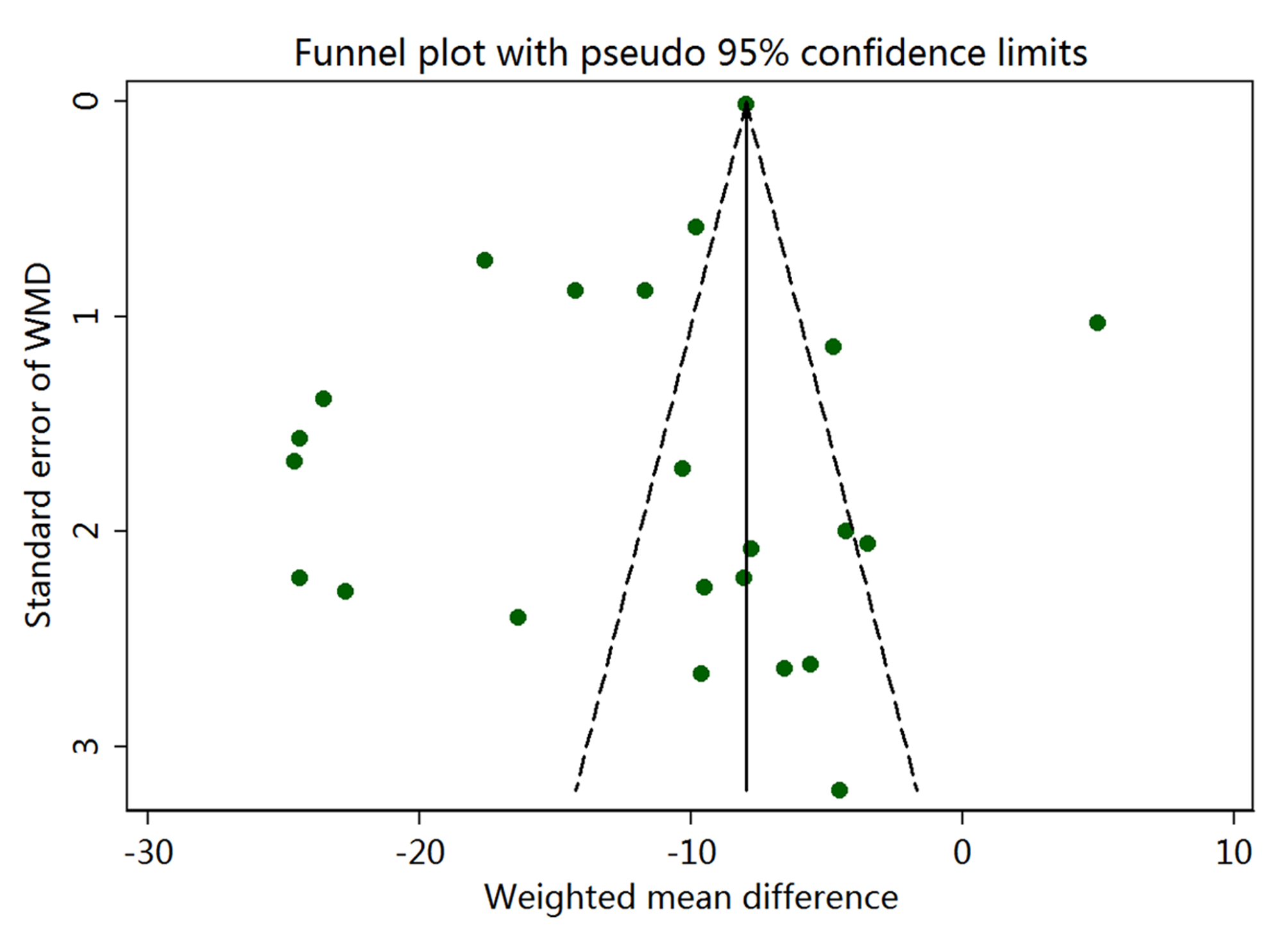

Supplement: Supplementary file 10 — Figure S10. Funnel plots demonstrating no significant publication bias among the included studies for WT in PAH. (TIF 360 kb) [file 13287_2019_1162_MOESM10_ESM.tif]
