# Supplementary material for: Efficacy of stem cell therapy for pulmonary arterial hypertension: a systematic review and meta-analysis of preclinical studies
Source: Stem Cell Res Ther. 2019 Feb 13;10:55. doi: 10.1186/s13287-019-1162-8 (PMC6374914; doi:10.1186/s13287-019-1162-8)
Supplement: Supplementary file 12 — Table S2. The detailed search strategy. (DOCX 17.6 kb) [file 13287_2019_1162_MOESM12_ESM.docx]

**Table S2. The detailed search strategy**

| **Electronic databases** | **Search** | **Search strategy** | **Results** |
| --- | --- | --- | --- |
| **Medline** | #1 | "Stem Cell"[Title/Abstract] | [136090](https://www.ncbi.nlm.nih.gov/pubmed/?cmd=HistorySearch&querykey=5) |
|  | #2 | "Stem Cell Research"[Mesh] OR "Stem Cell Niche"[Mesh] OR "Stem Cell Transplantation"[Mesh] OR "Cord Blood Stem Cell Transplantation"[Mesh] OR "Peripheral Blood Stem Cell Transplantation"[Mesh] OR "Mesenchymal Stem Cell Transplantation"[Mesh] OR "Cell Self Renewal"[Mesh] OR "Adult Stem Cells"[Mesh] OR "Stem Cells"[Mesh] OR "Hematopoietic Stem Cell Mobilization"[Mesh] OR "Hematopoietic Stem Cell Transplantation"[Mesh] | [240137](https://www.ncbi.nlm.nih.gov/pubmed/?cmd=HistorySearch&querykey=6) |
|  | #3 | #1 OR #2 | [287219](https://www.ncbi.nlm.nih.gov/pubmed/?cmd=HistorySearch&querykey=11) |
|  | #4 | "Pulmonary arterial hypertension"[Title/Abstract] | [10042](https://www.ncbi.nlm.nih.gov/pubmed/?cmd=HistorySearch&querykey=7) |
|  | #5 | "Familial Primary Pulmonary Hypertension"[Mesh] | [1831](https://www.ncbi.nlm.nih.gov/pubmed/?cmd=HistorySearch&querykey=10) |
|  | #6 | #4 OR #5 | [10410](https://www.ncbi.nlm.nih.gov/pubmed/?cmd=HistorySearch&querykey=12) |
|  | #7 | #3 AND #6 | [**156**](https://www.ncbi.nlm.nih.gov/pubmed/?cmd=HistorySearch&querykey=13) |
| **EMBASE** | #1 | 'stem cell':ab,ti | 201319 |
|  | #2 | 'stem cell'/exp | 316510 |
|  | #3 | #1 OR #2 | 403378 |
|  | #4 | 'pulmonary artery hypertension':ab,ti | 1870 |
|  | #5 | 'pulmonary hypertension'/exp | 84729 |
|  | #6 | #4 OR #5 | 84940 |
|  | #7 | #3 AND #6 | **1153** |
| **Cochrane Library** | #1 | (Pulmonary arterial hypertension):ti, ab, kw | 1644 |
|  | #2 | MeSH descriptor: [ Familial Primary Pulmonary Hypertension] explode all trees | 180 |
|  | #3 | #1 or #2 | 1676 |
|  | #4 | (Stem Cell):ti, ab, kw | 8800 |
|  | #5 | MeSH descriptor: [ Stem Cell] explode all trees | 749 |
|  | #6 | #4 or #5 | 8998 |
|  | #7 | #3 and #6 | **4** |
| **Web of Science** | #1 | TOPIC: ("stem cell") | 171,086 |
|  | #2 | TOPIC:("Pulmonary arterial hypertension") | 13,423 |
|  | #3 | #1 AND #2 | **29** |
